# Inactivation of the T6SS inner membrane protein DotU results in severe attenuation and decreased pathogenicity of *Aeromonas veronii* TH0426

**DOI:** 10.1186/s12866-020-01743-5

**Published:** 2020-04-03

**Authors:** Haichao Song, Yuanhuan Kang, Aidong Qian, Xiaofeng Shan, Ying Li, Lei Zhang, Haipeng Zhang, Wuwen Sun

**Affiliations:** College of Animal Science and Technology, Jilin Agricultural University, Changchun, Jilin, 130118 China

**Keywords:** *Aeromonas veronii*, Virulence, T6SS, Pathogenicity, *dotU*

## Abstract

**Background:**

The inner membrane protein DotU of *Aeromonas veronii* is an important component of the minimal core conserved membrane proteome required for the formation of an envelope-transmembrane complex. This protein functions in a type VI secretion system (T6SS), and the role of this T6SS during the pathogenic process has not been clearly described.

**Results:**

A recombinant *A. veronii* with a partial disruption of the *dotU* gene (720 bp of the in-frame sequence) (defined as ∆*dotU*) was constructed by two conjugate exchanges. We found that the mutant ∆*dotU* allele can be stably inherited for more than 50 generations. Inactivation of the *A. veronii dotU* gene resulted in no significant changes in growth or resistance to various environmental changes. However, compared with the wild-type strain colony, the mutant ∆*dotU* colony had a rough surface morphology. In addition, the biofilm formation ability of the mutant ∆*dotU* was significantly enhanced by 2.1-fold. Conversely, the deletion of the *dotU* gene resulted in a significant decrease in pathogenicity and infectivity compared to those of the *A. veronii* wild-type strain.

**Conclusions:**

Our findings indicated that the *dotU* gene was an essential participant in the pathogenicity and invasiveness of *A. veronii* TH0426, which provides a novel perspective on the pathogenesis of TH0426 and lays the foundation for discovering potential T6SS effectors.

## Background

*Aeromonas veronii*, an emerging opportunistic pathogen, is widely present in natural environments especially freshwater and estuaries [1, 2]. *A. veronii* can infect various aquatic organisms such as freshwater fish, shrimp, loach and turtles, as well as mammals including humans [3]. In general, *A. veronii* can reach its peak of reproduction in the summer and autumn seasons and aquatic organisms with surface damage and decreased immunity are more susceptible to infection. The clinical symptoms of aquatic organisms infected with *A. veronii* are different, and are characterized mainly by skin ulcers, bleeding from organs and severe ascites [4, 5]. To date, effective control of *A. veronii* infection is still hampered by the lack of sufficient understanding of the mechanisms involved in the pathogenesis and virulence of the bacterium.

The type VI secretion system (T6SS), as a contractile nanomachine, can not only transport toxins into other bacteria, but also puncture the envelope of eukaryotic cells to inject toxins [6, 7]. The T6SS is present in various bacteria, such as *Escherichia coli*, *Vibrio anguillarum*, *Salmonella enterica*, *Edwardsiella tarda* and *Pseudomonas aeruginosa*, and is commonly associated with enhancing bacterial adaptability to the external environment and mediating pathogenicity of bacteria to host cells [8–10]. Furthermore, T6SS has been reported to have toxic effects on cells [11, 12]. Recent reports show that T6SS is also involved in many other functions, such as the biofilm formation of *E. coli* and the stress response of *Vibrio anguillarum* [13, 14]. It can also promote symbiotic relationships between bacteria and eukaryotes and mediate cooperation as well as competition between bacteria. However, the biological functions of T6SS in the pathogenesis of *A. veronii* TH0426 infection remain poorly understood.

We recently described a novel *A. veronii* inner membrane protein encoding gene, *dotU* (namely *tssL*) [15], which is similar to the T6SS protein DotU in *Aeromonas hydrophila subsp. hydrophila* and *E. coli* [16, 17]. DotU is an inner membrane protein that form part of the membrane-joining complex of the T6SS. DotU binds tightly to IcmF and together they are tethered to the inner membrane at one end and the peptidoglycan layer at the other; they interact with Lip1 which then tethers the peptidoglycan layer to the outer membrane [18, 19]. However, there are no reports of the *dotU* gene being involved in bacterial growth, biofilm formation, oxidative stress resistance, flagellar integrity, pathogenicity, or virulence. Only the structure of the *dotU* gene and its involvement in the membrane complexes that are involved in stabilizing the T6SS device are reported in *Pseudomonas aeruginosa* and *Vibrio parahaemolyticus* and there is no specific description of the function of the *dotU* gene [20, 21]. Therefore, we attempted to explore the role of the *dotU* gene in pathogenic *A. veronii* to improve our understanding of TH0426 infection.

In the current study, we investigated the biological functions of the *dotU* gene in *A. veronii*. Therefore, a mutant of *A. veronii* with an in-frame deletion of the membrane-controlling gene *dotU* of the T6SS was constructed and a strain over-expressing the *dotU* gene was generated. We examined each of the response regulator mutants for growth, motility, biofilm formation, resistance to various environments, pathogenicity and infectivity. Our results initially reveal the role of the *dotU* gene in *A. veronii* and may provide new insight into the biological function of the DotU endomembrane protein of *A. veronii*.

## Results

### Sequence characterization of *dotU*

Whole-genome analysis revealed that the genome of *A. veronii* contains a complete T6SS, including Hcp, VgrG, IcmF, ClpV, DotU, VipA and VipB. *dotU* is located on the genome of the bacterium and extends 780 bp and encodes a 259-amino acid polypeptide with a calculated molecular weight of 30.2 kDa and a theoretical pI of 5.54. The primary structure of the DotU protein has no signal peptide, has a transmembrane region and is judged to be hydrophilic based on the previous evaluation standard. Secondary structure analysis shows that the DotU protein contains 37.8% random coil, 61% alpha helix, and 1.2% beta-sheet, making it primarily composed of alpha helix. DotU shares approximately 99, 98, 97, 96 and 94% overall sequence identity with the DotU family T6SS proteins of *Aeromonas sobria* and *Aeromonas dhakensis*, type VI secretion protein ImpK of *Aeromonas salmonicida*, and DotU family T6SS proteins of *Aeromonas enteropelogenes* and *Aeromonas eucrenophila*, respectively.

### Construction and confirmation of the mutant strain ∆*dotU* and complemented strain C-*dotU*

To investigate the biological roles of the *dotU* gene, a chromosomal mutant of *dotU* in *A. veronii* was constructed by allelic exchange that involved a deletion of 720 bp within the *dotU* open reading frame (ORF). The mutant and complemented strains were confirmed by DNA amplification (Additional file 1: Fig. S1a and b) and RT-PCR, respectively. For DNA amplification, gene fragment with sizes of 2932 bp and 606 bp were obtained, respectively, by using PCR amplification of the template genome DNA extracted from the wild-type, mutant and complemented strains. The results were consistent with the expectation that there would be no *dotU* gene signal in the mutant strain but that there would be a signal in the parent and C-*dotU* strains, which initially proved successful construction.

**Fig. 1:**
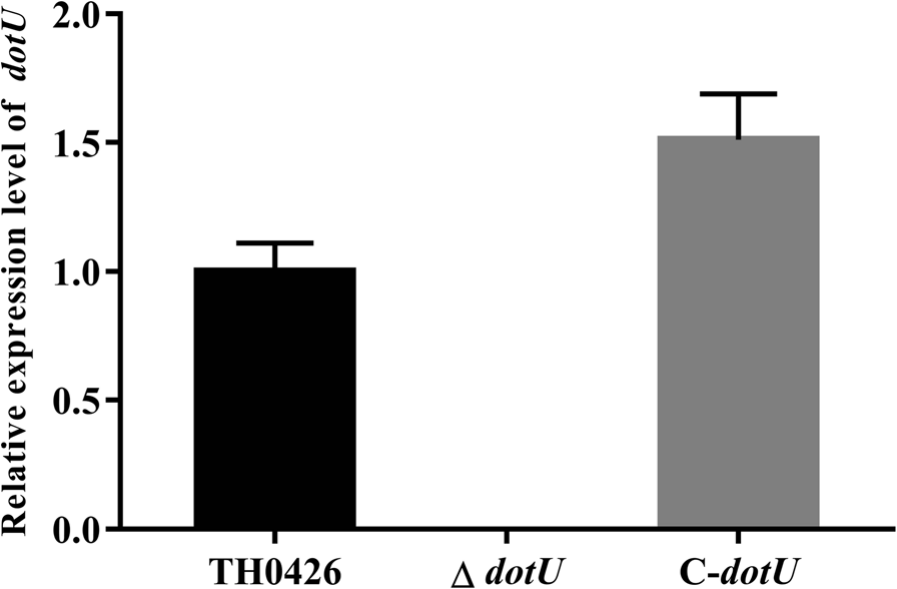
Relative expression level of the *dotU* gene in wild-type, mutant ∆*dotU* and complemented C-*dotU* strains. qRT-PCR was performed with total RNA extracted from three strains. Data are presented as means ± standard deviation (SD) (*n* = 3)

To further confirm the accuracy of the mutant strain ∆*dotU*, we extracted the total RNA of three strains. cDNA was obtained after reverse transcription and used as a template for further detection. The results also showed that *dotU* was inactivated successfully. Real-time PCR analysis further confirmed that *dotU* gene transcription could not be initiated in the mutant strain, but the transcriptional signal of this gene in the complemented strain was slightly higher than that in the wild-type strain (Fig. 1). In addition, the deletion and complemented mutations could be stably inherited for more than 50 generations (Additional file 1, Fig. S1c and 1d).

### Growth ability, colony morphology and haemolytic activity

**Fig. 2:**
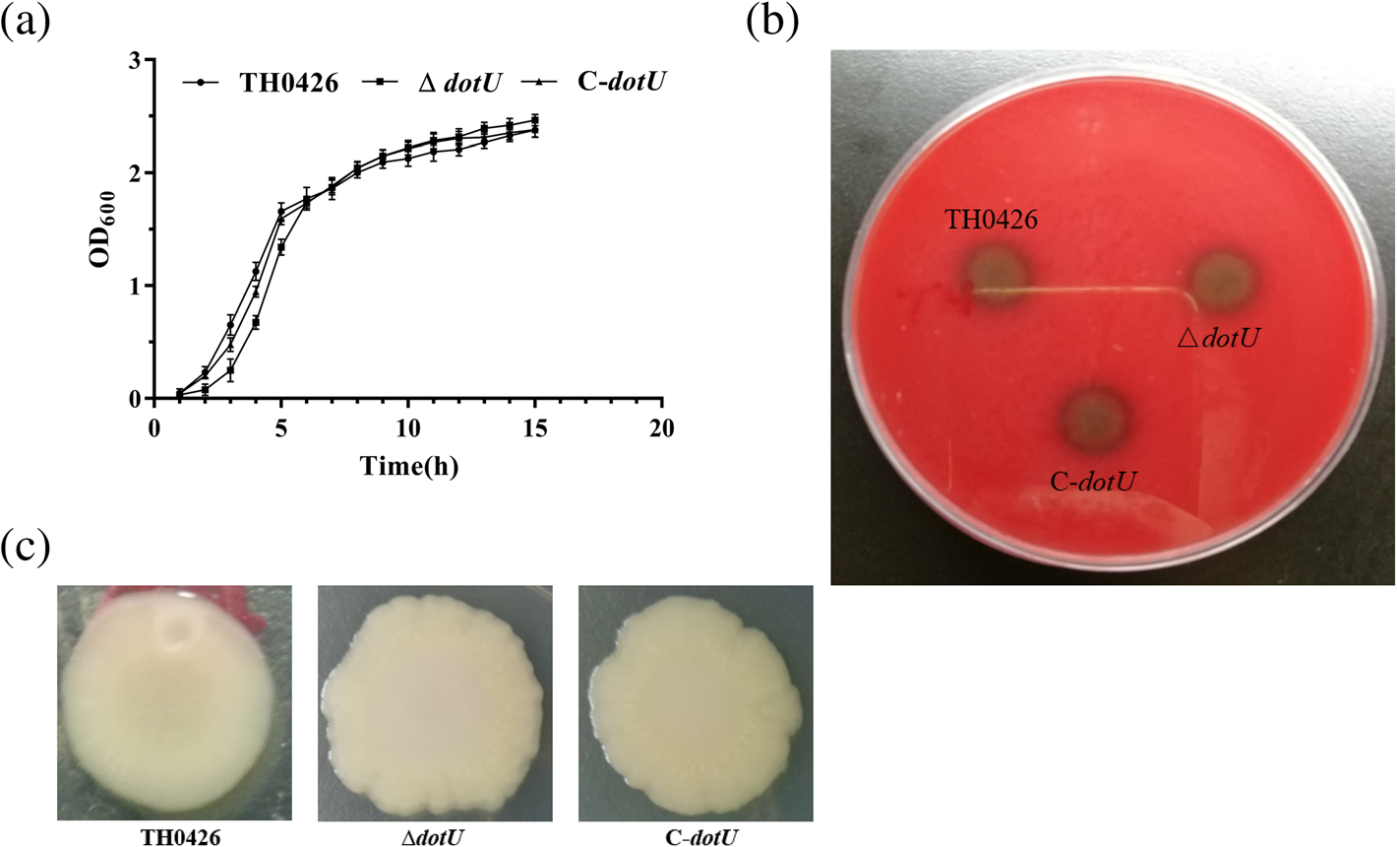
Effect of deletion of *dotU* gene from *A. veronii* on its growth, haemolytic activity and colony morphology. **a** Growth curves of wild-type strain TH0426, mutant strain ∆*dotU* and complemented strain C-*dotU* in LB at 30 °C with growth monitored by OD_600_. Values are showed as means ± standard deviations (SD) from as least three independent experiments. **b** Effect of *dotU* gene on haemolytic activity of *A. veronii*. **c** Changes in the surface morphology of colonies of the parental strain TH0426, mutant strain ∆*dotU* and complemented strain C-*dotU*

Growth analysis showed that the mutant ∆*dotU* exhibited a growth ability nearly similar to that of the parent strain and complemented strain (*P* > 0.05) during the logarithmic growth period and the growth yield of all strains is in a stable state with no difference during the stationary phase. In general, there was no obvious difference in the growth rate (Fig. 2a). In addition, the haemolytic activity of the three strains showed consistent results, all of which were beta-haemolysis positive (Fig. 2b). However, deletion of the *dotU* gene caused severe changes in *A. veronii* colony morphology with the emergence of undulating edges and a rough surface. These changes were rescued in the complemented strain, but the colony morphology of the complemented strain did not reach the original state (Fig. 2c).

### Flagellum formation and motility detection

**Fig. 3:**
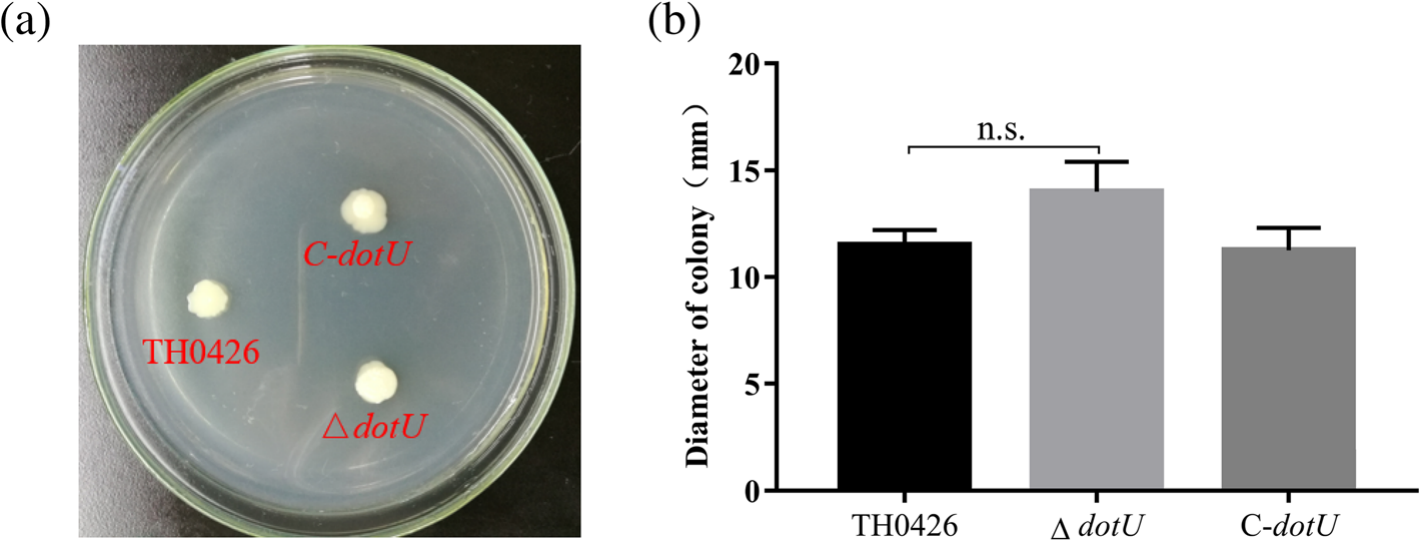
Changes of *dotU* gene deletion on motility ability of *A. veronii*. **a***A. veronii* has swimming ability. **b** The swimming diameter was detected to reflect changes in motility of wild-type TH0426, mutant strain ∆*dotU* and complemented strain C-*dotU* (n. s. indicating no significant difference)

After flagellm staining, light microscopy showed that there were clear, intact, single and long polar flagella in the staining field of the wild-type strain. A similar phenomenon prominently located on the end side of the bacterial body was found in both the deletion strain and the complemented strain (Additional file 1: Fig. S2). The swarming diameter showed no changes between the mutant and wild-type strains (*P* > 0.05) (Fig. 3).

### The *dotU* gene involvement in biofilm formation

**Fig. 4:**
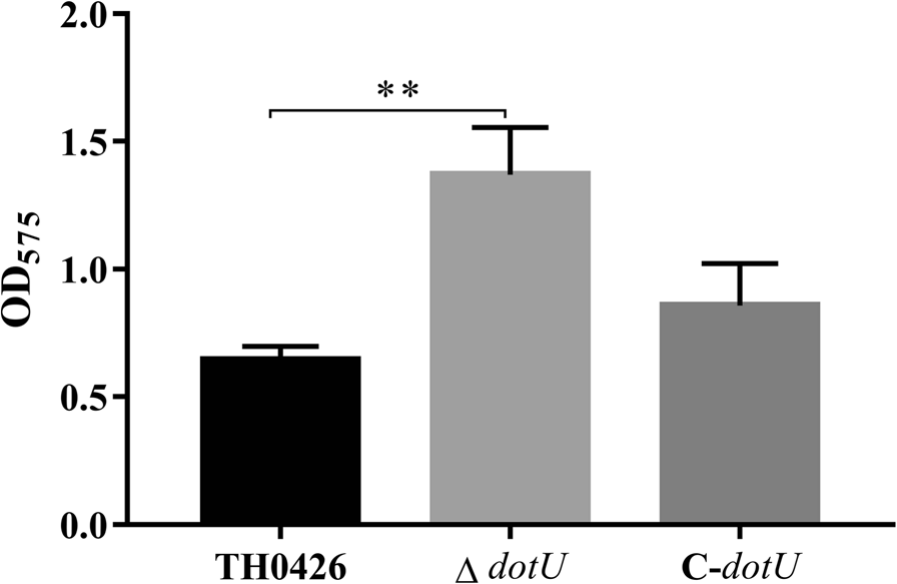
Biofilm formation ability of ∆*dotU*, C-*dotU* and parent strains. The amount of biofilm formation is presented by the OD_575_ value (** indicating *P* < 0.01)

The crystal violet staining microtiter biofilm assay was performed to investigate the effect of DotU on biofilm formation. The results reported that the OD_575_ values of the deletion strain ∆*dotU* and complemented strain C-*dotU* were 1.35 ± 0.15 and 0.87 ± 0.09, respectively, and the wild-type strain TH0426 and negative control had values of 0.64 ± 0.04 and 0.16 ± 0.03, respectively, which indicated that the biofilm formation ability of the mutant strain was 2.1-fold higher than that of the wild-type strain with a significant difference (Fig. 4), and that the biofilm formation ability was remarkably enhanced (*P* < 0.01).

### Differences in the LD_50_ of the 3 *A. veronii* strains

**Fig. 5:**
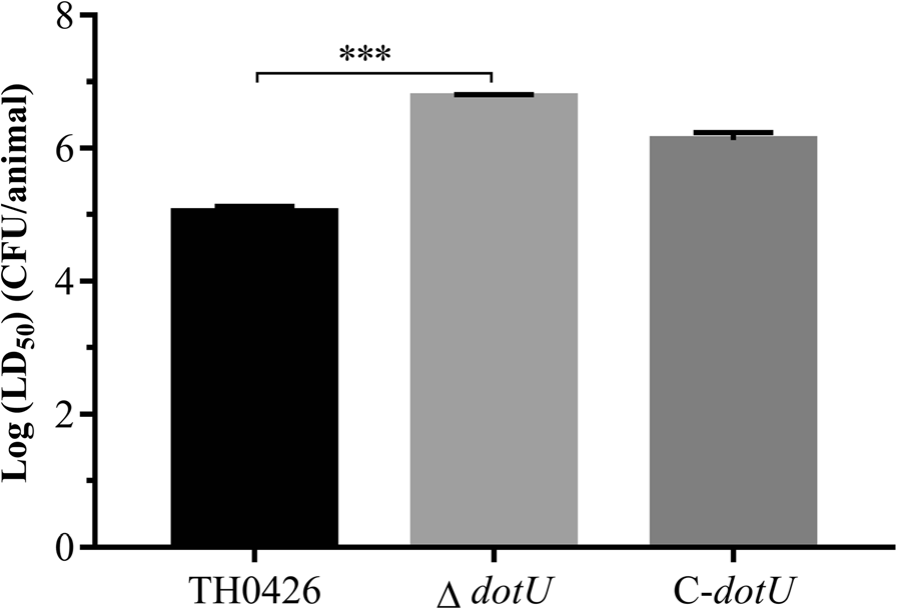
The half lethal dose (LD_50_) for adult zebrafish infected with *A. veronii*, mutant strain ∆*dotU* and complemented strain C-*dotU*. Data were presented as the logarithmic of LD_50_ (*** indicating *P* < 0.001)

To determine the virulence change in the *A. veronii dotU* deletion strain, the LD_50_ value was evaluated by establishing a zebrafish infection model. The results showed that the LD_50_ of the wild-type strain TH0426 for zebrafish was (1.25 ± 0.15) × 10^5^ CFU/tail, and that of the mutant strain Δ*dotU* was (6.3 ± 0.21) × 10^6^ CFU/tail; the virulence was therefore significantly decreased by 50.4-fold (*P* < 0.001). The LD_50_ of the complemented strain C-*dotU* was (1.6 ± 0.32) × 10^6^ CFU/tail, and the rescue effect was not significant (Fig. 5). The results indicated that deletion of the *dotU* gene caused severe attenuation of *A. veronii*.

### Epithelioma papulosum cyprini (EPC) cell adhesion ability

**Fig. 6:**
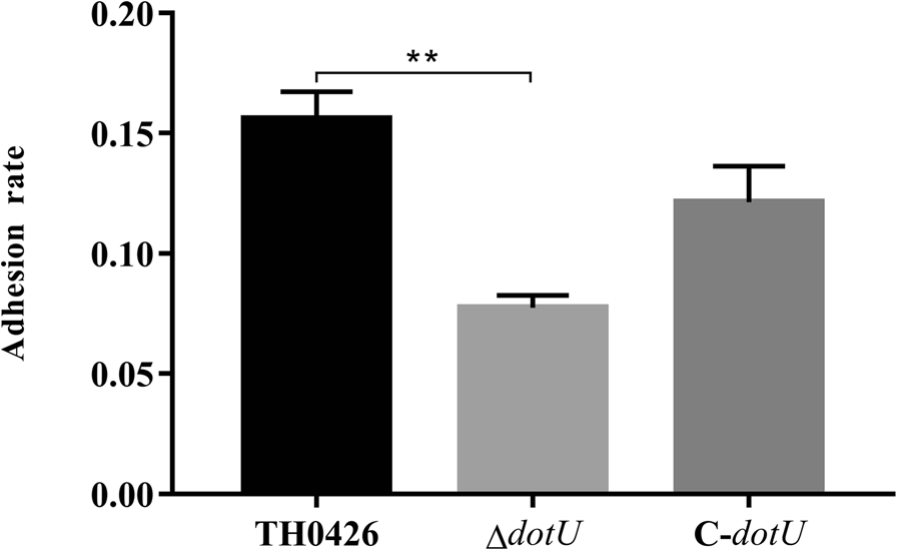
Evaluation of adhesion ability for ∆*dotU* mutant, complemented strain C-*dotU* and parent strain to EPC cells. Data were expressed as the adherence rate (** indicating *P* < 0.01)

The adhesion ability of the three strains were analysed to assess the effect of *dotU* gene deficiency on the virulence of *A. veronii,* the results are displayed as the ratio of the number of adherent bacteria to the number of initial colonies. The results showed that the adhesion rate of the deletion strain Δ*dotU* to EPC cells was 8% ± 0.9%, which was 2.0-fold lower than that of the wild-type strain 16% ± 1.1%. The difference was highly significant (*P* < 0.01), which indicated that the EPC cell adhesion ability of the mutant ∆*dotU* decreased significantly. Furthermore, compared with ∆*dotU*, the complemented strain C-*dotU* recovered slightly but did not reach the level of the wild-type strain (Fig. 6).

### The impaired cytotoxic effect of the *dotU* mutant

EPC cells were infected at 25 °C, and LDH release was evaluated at 30 min, 1 h and 2 h. At 30 min of infection, there was no significant change in the EPC cells. Over time, the cell morphology began to change at 1 h of infection, and all cells detached at 2 h. As shown in Table 1, the toxicity of the wild-type strain TH0426 to EPC cells was 1.8-fold higher than that of the deletion strain Δ*dotU* at 1 h of infection, the difference was significant (*P* < 0.01) and the virulence decreased significantly. At 2 h, the cytotoxicity reached a maximum of 2.1-fold more than that of the mutant strain; the difference was highly significant (*P* < 0.001), and the virulence was reduced significantly.

**Table 1:** Toxicity detection of mutant strain △*dotU* on EPC cells

	Cytotoxicity^a^ (%)	
Strain	1 h	2 h
TH0426	23.1 ± 0.33	68.3 ± 0.71
△*dotU*	12.5 ± 0.67**	32.7 ± 0.81***
C-*dotU*	18.2 ± 0.24	46.8 ± 0.51

Date are the means for three experiments and presented as mean ± SD

^a^Cell counts of bacteria that attach and invade EPC cell. *p < 0.05 (significant) versus corresponding values of TH0426

** and *** indicate *p* < 0.01 and *p* < 0.001 respectively

### Roles of *dotU* in the stress tolerance of *A. veronii*

**Fig. 7:**
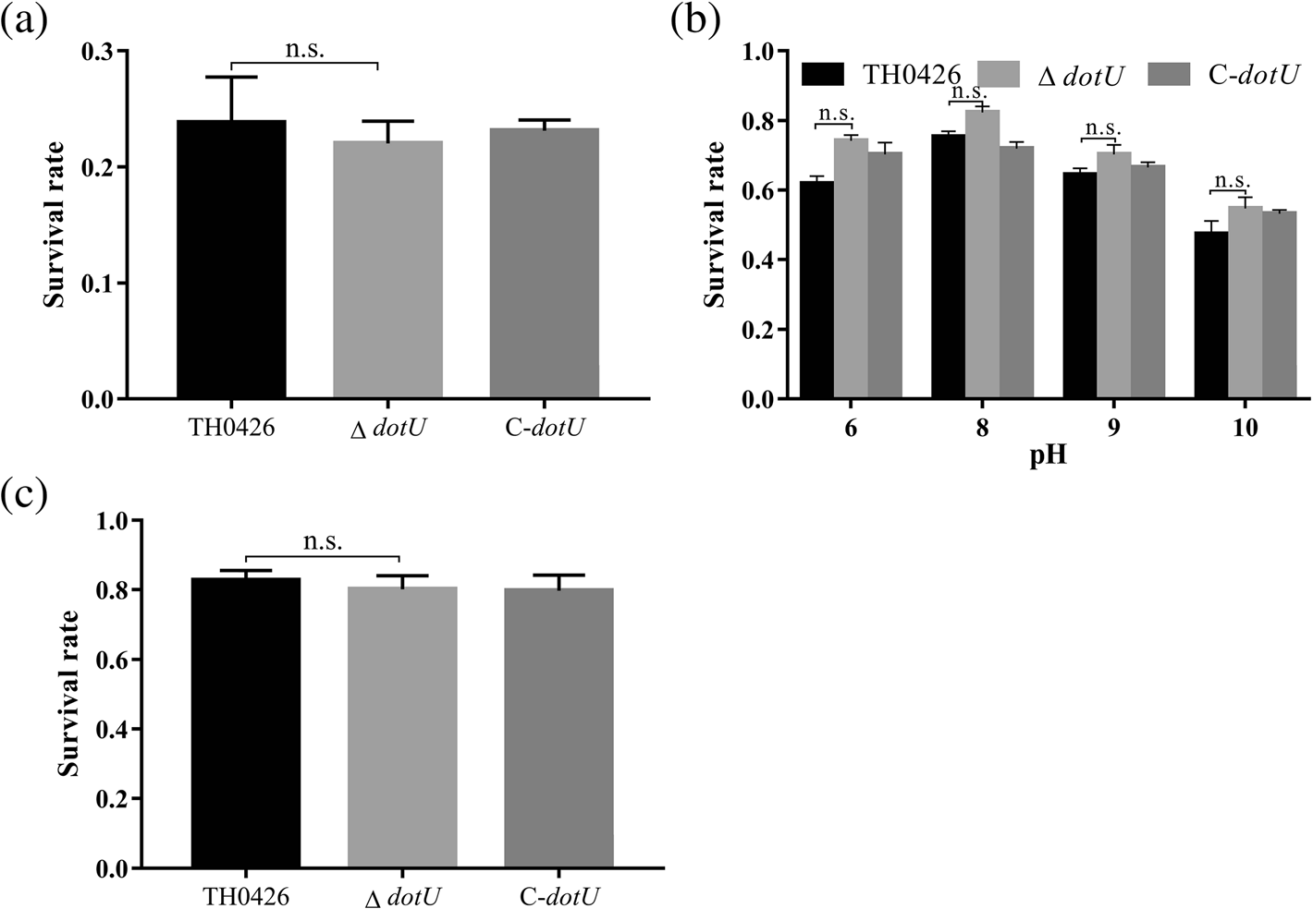
Susceptibility of *A. veronii* TH0426, ∆*dotU* and C-*dotU* to oxidative stress tolerance, pH tolerance and osmotic pressure tolerance. **a** Oxidative stress tolerance was detected by exposing logarithmic bacteria to a strong oxidizing environment (replaced with H_2_O_2_). **b** The pH tolerance of three strains by culturing in LB medium with a pH from 6 to 10 (the results of pH 7 are not shown). **c** Salt tolerance was analyzed by incubating the strains in LB medium containing 0.4 M NaCl

To evaluate the function of *dotU* in resisting environmental stress, the parent, mutant and complemented strains were exposed to a variety of stress challenges and we further detected the number of surviving bacteria of each strain using plate counting. When the wild-type strain in the pre-log phase was exposed to PBS containing 1 mM H_2_O_2_ for 1 h, the survival rate was 26.6% (Fig. 7a). The survival rates of the deletion strain Δ*dotU* and the complemented strain C-*dotU* were 25.4 and 26.2%, respectively, with no significant changes. Similar results were observed for resisting multiple environmental stresses (Fig. 7b and c), including osmolarity (NaCl, KCl), various pH values (6, 7, 8, 9 and 10) and thermal stress, which indicated that the T6SS membrane protein of *A. veronii* was not involved in the regulation of environmental stress.

## Discussion

Virulence factors are effector proteins that attach and are produced by bacteria to bring about the pathogenic role of bacteria. Bacterial pathogenesis can be initially revealed from the study of genes encoding these virulence factors, which can also be explored by studying the release mechanism of effector proteins. Studies have shown that *A. veronii* carries a large number of virulence factors [22], but since its discovery, the lack of recognition has resulted in the study of only known virulence factors as well as the isolation and identification of pathogenic strains [23]. The unknown virulence factors specifically involved in metabolic pathways, including those regulating gene networks, and controlling the pathogenicity of strains are rarely studied, which is increasingly becoming one of the obstacles to revealing the specific pathogenesis of *A. veronii*. The T6SS is a novel secretory mechanism that developed during the evolution of bacteria, and has a nanostructured phage-like injection structure that can inject effector proteins, i.e., virulence factors, into a host cell to exert toxic effects [24, 25]. The earliest discovered and most studied T6SS is in the genus *Vibrio* [26]. The *dotU* gene encodes the inner membrane protein of the T6SS. Its main function is to anchor the needle-like “syringe” structure of the T6SS to the surface of the cytoderm with the intimal protein IcmF and the outer membrane protein Lip. The C-terminal helix can enhance anchoring [27], which can facilitate stable and efficient injection of effector proteins into host cells to exert toxic effects [24, 28]. However, currently, gene deletion studies of the function of the *A. veronii dotU* gene have not been reported. Based on previous comparative proteomics and genomics analysis in the laboratory, we found that the expression level of the *dotU* gene in the virulent strain *A. veronii* TH0426 was significantly higher than that in an attenuated strain and an avirulent strain. We speculated that the *dotU* gene may be involved in the regulation of bacterial virulence and tolerance. Therefore, we used the gene knockout method to delete the *dotU* gene to construct the deletion mutant ∆*dotU* and its complemented strain and analysed the differences in colony morphology, drug sensitivity, growth status, physiological and biochemical characteristics, cell invasion and adhesion, biofilm formation ability, cytotoxicity, tolerance, motility ability, flagellar formation ability and oxidative stress-related gene expression. Then the possible functions of the *dotU* gene were initially evaluated in the pathogenesis of bacteria.

The deletion strain △*dotU* showed results similar to those of the parent strain with no significant changes in growth characteristics, motility capacity, flagellar formation ability, and haemolytic activity as well as physiological and biochemical attributes, drug sensitivity and tolerance (results not shown). Compared with the colony morphology observations for wild-type *A. veronii*, those of the deletion strain showed different results. △*dotU* deficient strains showed significant undulation on the edge of the colony with a roughened surface, and the wild-type strain TH0426 had a smooth surface and smooth edge. The difference between the two strains was significant. However, no relevant reports have been found and the specific reasons for these differences need to be further explored. The biofilm formation ability test showed that the mutant strain Δ*dotU* exhibited 2.1-fold greater biofilm formation than the wild-type strain TH0426, and that the biofilm formation ability was significantly enhanced. However, Hao Bin [29] found that the biofilm formation ability of *Vibrio anguillarum* decreased significantly with the lack of the endomembrane proteins IcmF and VasF in a study of the function and regulation of the *Vibrio anguillarum* T6SS. In their research, the T6SS of *V. anguillarum* was found to indirectly regulates the formation of biofilms by the σ38 factor. Contrary to the results of this test, there are some differences in the functions performed by related genes of the T6SS for different strains.

Tolerance-related experimental results showed that compared with the wild-type strain, the deletion strain Δ*dotU* showed no significant difference (results not shown). In a study of the stress function of the pseudobinding Yersinia T6SS, Weipeng et al. [30] found that T6SS expression can be induced by H_2_O_2_ and has antioxidant capacity. The T6SS can maintain the hydroxyl level in bacteria and reduce DNA damage to perform antioxidant functions. However, this research found that the antioxidant capacity of the strain did not change significantly after the deletion of the *dotU* gene. It may be that the overexpression of the intimal protein IcmF repaired the loss of the intimal protein DotU to a certain extent, or that *dotU* was not involved in the regulation of bacterial tolerance stress, which is subject to further research and confirmation. Yu Ying et al. [31] found that the growth, acid stress resistance, biofilm formation ability, and toxicity to cells of an *icmF* gene-deficient strain did not change significantly in their study of the T6SS. However, the adhesion ability of the deletion strain decreased significantly. Whether the cause of this result is related to the destabilization of the secretory device caused by the loss of the intimal protein control gene, remains to be further studied. There were some similar results in this experiment, and also some differing results. The specific pathogenic mechanism still needs to be further explored based on the corresponding experiments.

The animal pathogenicity test showed that the LD_50_ of the mutant strain Δ*dotU* increased 50.4-fold compared with that of the wild-type strain and the virulence was also significantly reduced. The toxicity test with EPC cells did not show significant changes in the early stage of infection, but the toxicity of the mutant strain was significantly lower than that of the wild-type strain TH0426 in the middle and late stages of infection, and led to cell lysis and death. In addition, the EPC cell adhesion and invasion ability of the deletion strain Δ*dotU* decreased significantly by 2.3-fold, but no significant change was observed in its motility. Similar to this result, Wang S et al. [32] found that the adhesion and colonization ability and resistance to-serum killing and phagocytosis of avian pathogenic *E. coli* significantly decreased. The production of the secreted protein Hcp1 was greatly limited. Jeanette E. Bro ¨ms et al. [33] obtained similar results in the study of *Francisella tularensis*. They found that the deletion strain Δ*dotU* could not escape from phagocytic cells and that virulence was significantly attenuated in a mouse model, showing no pathogenicity. Similar attenuation results were detected in this experiment, which indicated that some gram-negative bacteria may share the same T6SS. The specific pathogenesis may result from the deletion of the *dotU* gene mediating the *icmF* gene and affecting the stability of the intimal component, or from the two inner membrane proteins interacting to stabilize the secretory device of the T6SS, which are still subject to further proof.

## Conclusions

In summary, we systematically characterized the multiple effects of the *dotU* gene for the first time in *A. veronii* TH0426 and demonstrated that the *dotU* gene contributed to colony surface morphology, biofilm formation, pathogenicity and virulence in *A. veronii*. All of these phenotypes were recovered in the complemented strain. Deeper studies are required to promote an enhanced understanding of the inner membrane protein of the T6SS and the physiological traits that they impact.

## Methods

### Bacterial strains, plasmids and culture conditions

The wild-type strain *A. veronii* TH0426 used in this study was initially isolated from a farmed yellow catfish in Zhejiang Province, China. All *E. coli* in this experiment were cultured at 37 °C using Luria-Bertani (LB) solid or liquid medium. *A. veronii* was cultured at 28 °C using LB solid or liquid medium and *Aeromonas*-selective solid Rimler-Shotts (RS) medium. The pEASY-Blunt Zero (pEASY), suicide plasmid pRE112 and broad-host expression plasmid pBBR1-MCS, were used for gene amplification, conjugation with the genome of *A. veronii* and gene expression, respectively. When required, the concentrations of antibiotics were 100 μg/mL ampicillin (Amp) for *E. coli* and *A. veronii* and 50 μg/mL chloramphenicol for *E. coli*. The bacterial strains and plasmids used in this study and their relevant characteristics are listed in Table 2.

**Table 2:** Bacterial strains and plasmids used in this study

Strain or plasmid	Properties	Source or Reference
Strains		
*A. Averonii* TH0426	wild type isolated from the yellow catfish, Amp^r^	This study
∆*dotU*	Isogenic *dotU* mutant of strain TH0426	This study
C-*dotU*	Mutant ∆*dotU* complemented with intact *dotU* gene	This study
*E. coli*		
Trans1-T1	F-φ80(*lacZ*)ΔM15Δ*lacX*74hsdR (*rk -, mk +*) Δ*recA*1398endA1tonA	TransGene Biotech
DH5α-λpir	*Λpir*-lysogen of DH5α	Stored in our lab
WM3064	*thrB1004 pro thi rpsL hsdS lacZ△M15RP4–1360(araBAD)567△dapA1341::[erm pir(wt)]*	Stored in our lab
Plasmids		
pEASY-Blunt Zero	TA cloning vector, Amp^r^	TransGene Biotech
pEASY-UD *dotU*	Carrying the flanking region of the ORF for *dotU* TA cloning	This study
pRE112	pGP704 suicide plasmid, pir depengent, *oriT*, *oriV*, *sacB*, Cm^r^	Stored in our lab
pRE112-UD *dotU*	pRE112 carrying the flanking region of the *dotU* ORF, Cm^r^	This study
pBBR1-MCS	Broad-host-range vector, Cm^r^	Stored in our lab
pBBR-*dotU*	pBBR carrying of 998 bp containing the promoter and *dotU* ORF, Cm^r^	This study

### Ethics statement

Adult AB/TU wild red zebrafish (3–6 months old), purchased from a commercial fish market in Changchun City, China, were approximately 0.15 g of body weight and approximately 2.8 cm of body length. The experimental animals were kept in a clean, specific pathogen-free (SPF) barrier environment. All experiments were performed in strict accordance with the regulations of the Animal Care and Use Committee of Jilin Agriculture University (JLAU08201409) and the National Institutes of Health Guide for the Care and Use of Laboratory Animals (NIH Publications No.8023). All remaining experimental animals were euthanized by bringing the concentration of clove oil in the water to 80 mg/L. Collection of organ samples from the farmed yellow catfish complies with the Ethics Committee of Zhejiang Institute of Freshwater Fisheries.

### Sequence analysis

The nucleotide sequence of the *A. veronii dotU* gene was obtained from the *A. veronii* genome. The sequence of *dotU* was analysed using the BlAST program at the National Center for Biotechnology Information (NCBI) and the Expert Protein Analysis System. The DotU protein structure was analysed using the corresponding protein structure online prediction tools.

### Construction of an *A. veronii* mutant and complemented strain

To construct the *dotU* deletion mutant strain of *A. veronii*, ∆*dotU*, 720 bp of the *dotU* gene were disrupted. In short, the first and second PCRs were performed with *dotU*-f/*dotU*-int-r and *dotU*-int-f/*dotU*-r to amplify the upstream and downstream fragments of the *dotU* gene, respectively. The two purified flanks were then ligated by fusion PCR, and inserted into a linear vector, pRE112, digested at the same restriction site, named pRE112-UD *dotU*. The resultant suicide vector was then transformed into the gene-engineering strain WM3064. After that, WM3064 and *A. veronii* were co-cultured to induce homologous recombination. The transconjugants that underwent the first homologous recombination were selected on LB agar plates supplemented with Amp and Cm. Subsequently, double crossover (DC) recombination was induced on plates containing Amp and 10% sucrose to spontaneously achieve suicide vector excision from the genome. All the primers for the construction of the corresponding strains are listed in Table 3.

**Table 3:** Primers used in this study

Primers	Nucleotide sequence
*dotU*-f (Xba I)	CGTCTAGAGCCGAGGTGACCGAGGCGACCATGG
*dotU*-int-r	**GGAACTTCTG**GCTGCTTCTTTGTACTGATTT
*dotU*-int-f	**AAGAAGCAGC**CAGAAGTTCCGCGCCCATT
*dotU*-r (Sac I)	TAGAGCTCCCGAGAGCTTGAGGGTGCCGT
*dotU*-e-f	GCGCCATGGACGAAGTTGACC
*dotU*-e-r	ATCCGCAGCGCCAGATCGAA
*dotU*-i-f	AGGCCACACCCACCACAGGCT
*dotU*-i-r	CTGCAAACCCTGGCGGCTCC
*dotU*-p-f (Pst I)	AACTGCAGTTTCCATCCTCAGTAAATAATGA
*dotU*-p-int-r	**TCAGTACAAA**CCGTTCGTTCCTTCATCTT
*dotU*-O-f	**GAACGAACGG**ATGGGCGCGGAACTTCTGC
*dotU*-O-r (Hind III)	GCAAGCTT TCAGTACAAAGAAGCAGCAGGCC
16S-q-f	GCCACGTCTCAAGGACACAG
16S-q-r	TGGGGAGCAAACAGGATTAGA
*dotU*-q-f	TCTATGGCATACCGGAAAAGG
*dotU*-q-r	ACTGAACACCGCAAAGAGCA
pRE112-f	GCGATGAGTGGCAGGGC
pRE112-r	TTACCGACTGCGGCCTGAGT
pBBR-f	TAAGTTGGGTAACGCCAGG
pBBR-r	GAGTTAGCTCACTCATTAGGC

A promoter was accurately screened to drive the expression of the *dotU* gene and carried by the *dotU* gene to be amplified using primers *dotU*-O-f/*dotU*-O-r. Then, the retrieved PCR product was ligated into the broad-host expression plasmid pBBR1-MCS of gram-negative bacteria to construct the expression plasmid pBBR-*dotU*. The plasmid was then introduced into the *dotU* mutant to construct the complemented strain. PCR and RT-PCR were used to detect whether the mutant and complemented strains were successfully constructed.

### Growth characteristics, morphology and haemolysis activity

According to a previous method with some modifications, the growth curve of the bacterial strains was monitored by determining the OD_600_ value of each bacterial culture. Briefly, for colony counting, the three strains (wild-type *A. veronii*, mutant ∆*dotU* and complemented strain C-*dotU*) were cultured overnight for approximately 12 h. Then, the bacterial concentration was adjusted to the same starting point and the OD_600_ value was determined. Then the same density of the three bacterial suspensions was inoculated onto an LB agar plate and cultured for 48 h. The bacterial colony morphology was observed by Gram staining. The same volume of bacterial suspension was transferred to a new 50 mL conical flask containing liquid LB and cultured at 28 °C with 180 rpm shaking, which was collected at intervals of 1 h. The OD_600_ value was determined, and the growth was recorded. The same volume of bacterial suspension was inoculated onto a sheep blood plate and cultured in a 28 °C incubator for 24 h to observe lytic activity. All experiments were performed at least 3 times.

### Flagella formation and motility test

Flagellar staining of *A. veronii*, the deletion strain ∆*dotU* and the complemented strain C-*dotU* was performed using a flagellar staining kit (modified Ryu method, Solarbio Inc., Beijing). Then, the flagellar synthesis of the corresponding strains was observed by light microscopy, according to the kit instructions for specific test procedures. The swimming and swarming abilities were detected by LB containing 0.3% agar, 0.5% agar, and 5% glucose at 28 °C for 24 h. Then the distances were measured later. Both experiments were repeated at least three times.

### Biofilm assay

Based on the biofilm formation ability detection method used by Watnick PI et al. [34], the 96-well plate method was used to detect the biofilm formation ability of the bacteria using the biofilm formation conditions of *A. veronii* with appropriate improvements. Briefly, overnight culture supernatant was removed, and the wells were gently rinsed 3 times with fresh phosphate-buffered saline (PBS). The attached cells were fixed with 99% methanol for 20 min, and after discarding, the wells were stained with 2% crystal violet dye solution for 10 min. After drying, the crystal violet was dissolved with 33% acetic acid and fully mixed. Ten replicates were set up per bacterium in each experiment to detect biofilm formation, and the assay was performed at least three times. A previous report [35] showed that *A. veronii* TH0426 was an efficient biofilm-producer and the evaluation standard of biofilm formation ability was performed as follows. i: when the OD_575_ value of the experimental group was less than or equal to that of the negative control group (OD_575N_), there was no biofilm formation ability (negative); ii: OD_575N_ < OD_575_ ≤ 2OD_575N_, 2OD_575N_ < OD_575_ ≤ 4OD_575N_ and OD_575_ > 4OD_575N_ were judged to be weaker, medium and stronger biofilm formation abilities, respectively; and iii: the OD_575_ value was exhibited by the mean (mean) ± standard deviation (SD) of three experiments.

### Median lethal dose (LD_50_) in the zebrafish model

The LD_50_ values of all strains were determined in zebrafish as described previously to assess the pathogenicity of the three strains (*A. veronii* TH0426, ∆*dotU* and C-*dotU*). Bacterial culturing, colony counting and concentration adjustment were performed according to the above method. The bacterial liquid was diluted 10-fold with sterile PBS and set to 8 gradients. Then healthy zebrafish were divided into twenty-four groups with ten fish in each tank. Each of the fish was injected intraperitoneally with a 10 μL bacterial solution and the control group was treated with an equal volume of PBS. The mortalities were recorded over 10 days after infection and moribund fish were removed and inspected visually for the status of the disease. Then LD_50_ values were calculated by Kou’s law [36].

### Adhesion ability of EPC cells

The detection of adhesion ability which was improved by referring to the relevant method reported previously [37], was conducted to analyse the adhesion ability of the three strains (*A. veronii*, the mutant ∆*dotU* and the complemented strain C-*dotU*). In brief, EPC [38] cells were cultured in M199 medium containing 10% heat-inactivated foetal bovine serum and 1% double antibiotic (penicillin and streptomycin) at 25 °C in an incubator with 5% CO_2_. EPC monolayers were grown for 24 h in 24-well tissue culture plates and infected by the three strains, which were washed and resuspended with M199 at a multiplicity of infection of 10:1. The same volume of sterile PBS was used as a control. After infecting cells for 1 h, the cells were washed several times with PBS and then lysed with 1% Trion X-100 at 25 °C for 1 h. The cell lysate was mixed thoroughly and diluted properly, and then the number of bacteria was quantified by plate counting.

### Cytotoxicity test

Based on related research [39], lactate dehydrogenase was used as a marker to evaluate the cytotoxicity of bacteria. Here, we evaluated the toxicity of the three strains to EPC cells according to the instructions of the cytotoxic kit. The infection status was observed to determine the optimal time of cytotoxicity detection. Finally, cytotoxicity was displayed by calculating the lactate dehydrogenase (LDH) release percentage for each strain.

### Tolerance test

The stress resistance of the three strains was detected as described previously with some modifications [40, 41]. Briefly, an overnight culture was adjusted for concentration and then treated as follows. For the oxidative stress test: the washed cells were resuspended in 1 mL of sterile PBS containing 1 mM hydrogen peroxide at 28 °C with 180 rpm shaking for 1 h. For the pH tolerance test: the bacteria were suspended in 1 mL of sterile PBS at pH values of 3, 4, 5, 6, 8 and 10 and placed at 28 °C with 180 rpm shaking to culture for 1 h. For the heat stress tolerance test, the cells were resuspended in 1 mL of sterile PBS and incubated in 55 °C water for 1 h. For the osmotic pressure tolerance test, 1 mL of a bacterial solution in the logarithmic growth phase was serially diluted and then directly coated on solid LB plates containing 0.4 M NaCl. After that, the bacterium-coated plate was incubated overnight in a 28 °C incubator, and the colonies were counted. All tolerance tests contained a bacterial population resuspended in 1 mL of sterile PBS for 1 h at 28 °C that was used as a blank control. Each tolerance test was repeated three times in parallel.

### Statistical analysis

The data were analysed with one-way analysis of variance (ANOVA) followed by Duncan’s new multiple range test and Tukey’s test with SPSS 13.0 software. A significant difference was considered at ^*^P < 0.05 and ^**^P < 0.01.

## Supplementary information

**Additional file 1: Figure S1.** Confirmation of the success and genetic stability of mutant strain ∆*dotU* and complemented strain C-*dotU.* (a) PCR detection of the mutant strain ∆*dotU*, M: DL5000 Marker; 1: the mutant strain ∆*dotU*; 2: wild type TH0426. (b) PCR detection of the complemented strain C-*dotU*, M: DL2000 Marker; 1: C-*dotU*; 2: TH0426. (c) Genetic stability of the partial deletion strain ∆*dotU*, M: DL5000 Marker; 1–10: ∆*dotU*; 11–12: TH0426; 13: control group. (d) Genetic stability of the partial complemented strain C-*dotU*, M: DL2000 Marker; 1–10: C-*dotU*; 11–12: TH0426; 13: control group. **Figure S2.** Flagellum straining and light microscopy observation of the three strains (the wild-type, ∆*dotU* and C-*dotU*). Light microscopy images of parental strain (A), ∆*dotU* (B) and C-*dotU* (C). Magnifications, 1000 × (A, B and C).

## Data Availability

The datasets used and analysed during the current study are available from the corresponding author on reasonable request. All data generated or analysed during this study are included in this published article.

## Abbreviations

Amp: Ampicillin
BLAST: Basic Local Alignment Search
cDNA: Complementary Deoxyribonucleic acid
Cm: Chloramphenicol
DC: Double crossover
EPC: Epithelioma papulosum cyprini
LB: Luria-Bertani
LD_50_: Median lethal dose
LDH: Lactate dehydrogenase
NCBI: National Center for Biotechnology Information
OD600, OD575: Optical density
ORF: Open reading frame
PBS: Phosphate Buffered Saline
PCR: Polymerase Chain Reaction
pEASY: pEASY-blunt Zero
RNA: Ribonucleic Acid
RS: Rimler-Shotts
RT-PCR: Reverse Time-Polymerase Chain Reaction
SPF: Specific pathogen-Free
T6SS: Type VI secretion system
