# Supplementary material for: Inactivation of the T6SS inner membrane protein DotU results in severe attenuation and decreased pathogenicity of Aeromonas veronii TH0426
Source: BMC Microbiol. 2020 Apr 3;20:76. doi: 10.1186/s12866-020-01743-5 (PMC7119292; doi:10.1186/s12866-020-01743-5)
Supplement: Supplementary file 1 — Additional file 1: Figure S1. Confirmation of the success and genetic stability of mutant strain ∆dotU and complemented strain C-dotU. (a) PCR detection of the mutant strain ∆dotU, M: DL5000 Marker; 1: the mutant strain ∆dotU; 2: wild type TH0426. (b) PCR detection of the complemented strain C-dotU, M: DL2000 Marker; 1: C-dotU; 2: TH0426. (c) Genetic stability of the partial deletion strain ∆dotU, M: DL5000 Marker; 1–10: ∆dotU; 11–12: TH0426; 13: control group. (d) Genetic stability of the partial complemented strain C-dotU, M: DL2000 Marker; 1–10: C-dotU; 11–12: TH0426; 13: control group. Figure S2. Flagellum straining and light microscopy observation of the three strains (the wild-type, ∆dotU and C-dotU). Light microscopy images of parental strain (A), ∆dotU (B) and C-dotU (C). Magnifications, 1000 × (A, B and C). [file 12866_2020_1743_MOESM1_ESM.docx]

(a) (b)


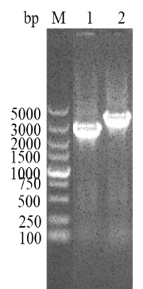

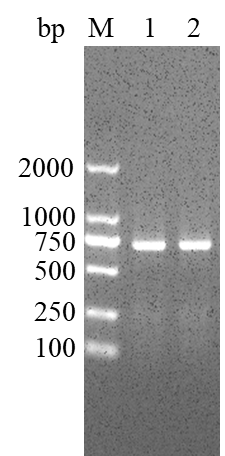


(c) (d)


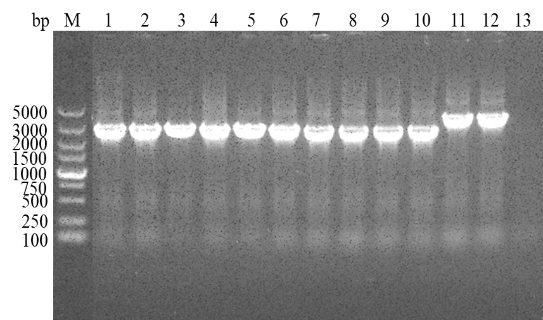

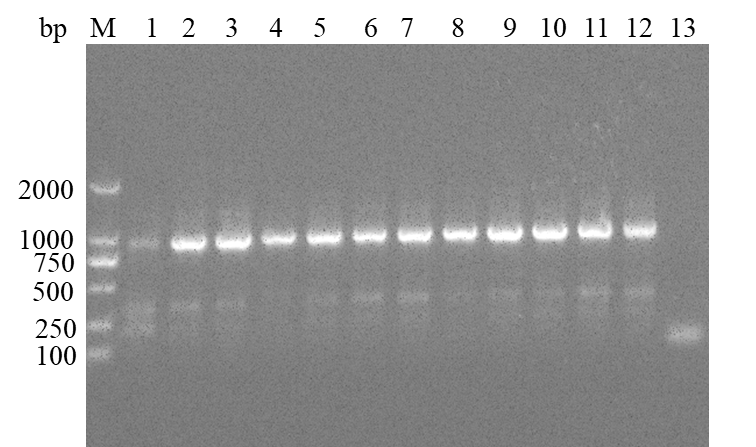


**Fig. S1 Confirmation of the success and genetic stability of mutant strain ∆*dotU* and complemented strain C-*dotU.*** (a) PCR detection of the mutant strain ∆*dotU*, M: DL5000 Marker; 1: the mutant strain ∆*dotU*; 2: wild type TH0426. (b) PCR detection of the complemented strain C-*dotU*, M: DL2000 Marker; 1: C-*dotU*; 2: TH0426. (c) Genetic stability of the partial deletion strain ∆*dotU*, M: DL5000 Marker; 1-10: ∆*dotU*; 11-12: TH0426; 13: control group. (d) Genetic stability of the partial complemented strain C-*dotU*, M: DL2000 Marker; 1-10: C-*dotU*; 11-12: TH0426; 13: control group.


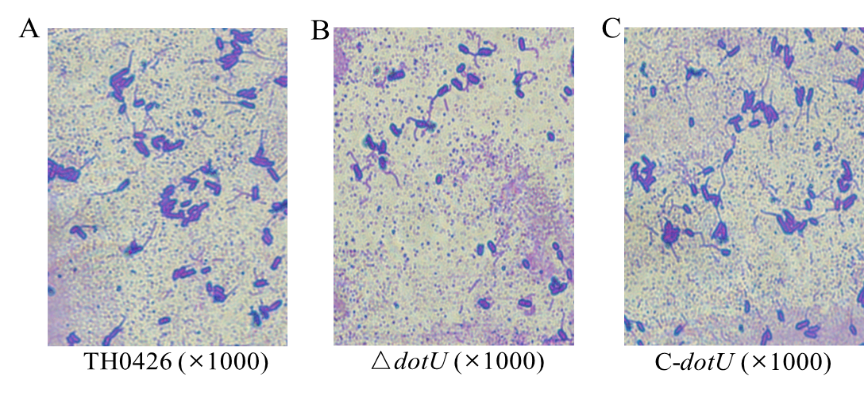


**Fig. S2 Flagellum straining and light microscopy observation of the three strains (the wild-type, ∆*dotU* and C-*dotU*).** Light microscopy images of parental strain (A), ∆*dotU* (B) and C-*dotU* (C). Magnifications, 1000 × (A, B and C).
